# Liver trauma in children: Our experience

**DOI:** 10.4103/0971-9261.43020

**Published:** 2008

**Authors:** Chrysostomos Kepertis, Athanasios Zavitsanakis, Antonios Filippopoulos, Konstantinos Kallergis

**Affiliations:** Department of Pediatric Surgery, Aristotle University of Thessaloniki, General Hospital “Georgios Gennimatas”, Thessaloniki, Greece

**Keywords:** Children, liver trauma

## Abstract

**Aims and Objectives:**

The purpose of this study was to determine the percentage, etiology, associated injuries and outcome of the children with liver trauma.

**Materials and Methods:**

In a retrospective review all children below 15 years of age who had sustained abdominal trauma were included. the study duration was January 1994 to December 2004.

**Results:**

Out of a total number of 436 trauma patients 34 were identified to have liver trauma (including one death). The median age was 5.89 and range: 1 to 14 years). Boys accounted for 76.4% (*n* = 26), and the most common cause was motor vehicle injuries, accounting for 41.17% (*n* = 14). Nine children underwent surgery (26.4%). Head injuries were the most common associated injuries, and the mortality rate was 2.94% (*n* = 1).

**Conclusions:**

The liver remained the second most commonly injured intraabdominal organ and nonoperative management is the preferred treatment for hemodynamically stable patients.

## INTRODUCTION

Trauma remains the leading cause of mortality in the pediatric population.[1–3] Blunt abdominal trauma is the major cause of abdominal injury in children. Nonoperative management of blunt hepatic trauma is the preferred treatment for hemodynamically stable patients.

## MATERIALS AND METHODS

A retrospective case note review was conducted for patients less than 15 years old with liver trauma admitted to the Department of Paediatric Surgery of Aristotle University of Thessaloniki. Data were collected on age, sex, mechanism of trauma, liver trauma, associated injuries, management and outcome. Liver injuries were graded according to the American Association for the Surgery of Trauma [Table 1].[1] Data were collected from January 1994 to December 2004. All the cases of abdominal trauma were reviewed.

**Table 1:** Liver injury scale classification

Grade	Injury description
I	
Hematoma	Subcapsular, nonexpanding, <10% of surface area
Laceration	Capsular tear, non bleeding, with <1 cm deep parenchymal disruption
II	
Hematoma	Subcapsular, nonexpanding, hematoma 10-50%, intraparenchymal, nonexpanding, <2 cm in diameter
Laceration	<3cm of parenchymal depth, <10 cm in length
III	
Hematoma	Subcapsular, >50% of surface area or expanding; ruptured subcapsular hematoma with active bleeding; intraparenhymal hematoma >2 cm
Laceration	>3 cm of parenchymal depth
IV	
Hematoma	Ruptured central hematoma
Laceration	Parenchymal destruction >75% of hepatic lobe
V	
Laceration	Parenchymal destruction >75% of hepatic lobe
Vascular	Juxtahepatic venous injuries (retrohepatic cava/major hepatic veins)
VI	
Vascular	Hepatic avulsion

## RESULTS

Out of 436 children admitted with abdominal injuries 34 (7.8%) sustained liver injuries. Boys accounted for 76.4%, including one death. The median age was 5.89 years and the age range was 11 months-14 years. The only death (2.94%) that occurred was of a five-year-old boy. He had uncontrollable bleeding during surgery following an assault injury. Motor vehicle injuries (MVI) were the mechanism of injuries in 41.17% of all the cases. Nine children (26.4%) underwent surgery for liver injuries and/or other related injuries [Table 2].

**Table 2:** Demographic profile of patients with liver injuries

Age	Sex	Mechanism of injury
Median (year): 5.89	Male: 76.4% (26)	MVI: 41.17% (14)
Range: 11 months-14 years	Female: 23.6% (8)	Fall: 23.5% (8)
		Assaults: 17.6% (6), including 1 death
		Pedestrian: 11.7% (4)
		Bicycle: 5.88% (2)

Abdominal ultrasound was the most common type of imaging (53%) in the first half of the study and abdominal CT (60%) was the most common imaging in the second half of the study.

Computed tomography or laparotomy demonstrated an isolated injury to the right lobe in 19 cases, the left lobe in eight cases, both the left and right lobe in five cases and a falciform ligament injury in two cases. Liver injury scores were graded according to the scale established by the American Association for the Surgery of Trauma [Table 1]. Nine patients were found to have grade I injuries; eight, grade II; nine, grade III, five, grade IV; two, grade V and one, grade VI [Table 3].

**Table 3:** Liver injury scores (LIS) and number of children

LIS	No. of children (*n* = 34) (%)	No. of deaths (*n* = 1)	No. of patients who underwent surgery (*n* = 9)
I	9-26.4	0	0
II	8-23.5	0	1
III	9-26.4	0	1
IV	5-14.7	0	4
V	2-5.88	0	2
VI	1-2.94	1	1

From the nine children that underwent surgery, only four (two grade IV, one grade V and one grade VI) were taken for laparotomy with liver injury being the only indication. The remaining five patients had other injuries including two splenic injuries, two head injuries, one fracture and one diaphragmatic injury [Table 3]. The decision to operate was based on the haemodynamic instability following volume and blood resuscitation (requirement >40 ml/kg blood).

Of the total number of cases, eight had an isolated hepatic injury and 26 suffered multiple injuries. There were eight head injuries, six thoracic injuries, four fractures, four spleen injuries, two kidney injuries, one pancreatic injury and one diaphragmatic injury [Table 4].

**Table 4:** Associated injuries

Head injuries	8
Thoracic injuries	6
Fractures	4
Spleen injuries	4
Kidney injuries	2
Pancreatic injuries	1
Diaphragmatic injuries	1

Once the diagnosis was confirmed, the patients were closely monitored in a high dependancy unit. There were no complications in the nonoperative group, whereas in the operative group (*n* = 4), two of them required a second-look operation after tamponade of the bleeding with laparotomy pads and removal of the packing. The other two patients required a nontypical excision part of the liver (one death).

The average length of admission was 10 days (range: 3 to 22 days). Four (11.7%) children required admission to the intensive care unit.

## DISCUSSION

Trauma is the leading cause of death and disability in children[2,3] worldwide and this agrees with our experience at the Department of Paediatric Surgery of Aristotle University of Thessaloniki. Blunt liver trauma is uncommon, but it is associated with significant morbidity and mortality. Even though our mortality rate was 2.94%, many severely injured children died before admission to hospital. The liver is the second most commonly injured intraabdominal organ and the right lobe is injured more frequently than the left, probably because of its size.[4,5] In our study, spleen is the first most commonly injured intraabdominal organ.

In agreement with the other centres, the reason of the injuries of the majority of our patients is motor vehicle accidents[6,7] accounting for 41.17%. Blunt abdominal trauma was the only etiology in our series. Head injuries were the most common associated injuries. Surgery is considered for isolated liver injury if the volume replacement in the first few hours after injury is more than 40 ml/kg.[8–10] In our study, length of admission is 10 days instead of 5-7 days according to other studies.[8,11] We believe that the difference is in the first 5-year period, where our treatment was an early operative approach. Our patients were advised to abstain from sport activities for 3 months for cases with nonoperative management and 6 months for the cases that underwent surgery.[8,10,12]

Ultrasound imaging was chiefly reserved for follow-up monitoring;[13] however, only a few patients returned for follow-up.

Patients with blunt liver injuries must be treated in a centre with pediatric surgeons available even though most patients could be treated nonoperatively. The challenges are the early identification of the severely injured child and the initiation of aggressive resuscitation.[13,14]

## References

[1] Moore EE, Cogbill TH, Jurkovits GJ, Shackford SR, Malangoni MA, Champion HR (1995). Organ injury scaling: Spleen and liver (1994 revision). J Trauma.

[2] Meyer AA (1998). Death and disability from injury: A global challenge. J Trauma.

[3] Gaines BA, Ford HR (2002). Abdominal and pelvic trauma in children. Crit Care Med.

[4] Bond SJ, Eichelberger MR, Gotschall CS, Sivit CJ, Randolph JG (1996). Non operative management of blunt hepatic and splenic injury in children. Ann Surg.

[5] Eichelberger M, Mooney D, Ziegler M, Azizkhan R, Weber T (2003). Abdominal trauma in operative pediatric surgery.

[6] Parks RW, Chrysos E, Diamond T (1999). Management of liver trauma. Br J Surg.

[7] Cooper A, Barlow B, DiScala C, String D (1994). Mortality and truncal injury: The pediatric perspective. J Pediatr Surg.

[8] (2005). Advanced life support group. Advanced paediatric life support. The practical approach.

[9] Stylianos S (2000). Evidence-based guidelines for resource utilization in children with isolated spleen or liver injury The APSA Trauma Committee. J Pediatr Surg.

[10] Losty PD, Okoye BO, Walter DP, Turnock RR, Lloyd DA (1997). Management of blunt liver trauma in children. Br J Surg.

[11] Coughlin PA, Stringer MD, Lodge JPA, Pollard SG, Prasad KR, Toogood GJ (2004). Management of blunt liver trauma in a tertiary referral centre. Br J Surg.

[12] Kumar R, Holland AJA, Shi E, Cass DT (2002). Isolated and multisystem hepatic trauma in children: The true role of non-operative management. Pediatr Surg Int.

[13] Landau A, van As AB, Numanoglu A, Millar AJ, Rode H (2006). Liver injuries in children: The role of selective non-operative management. Injury.

[14] Bond SJ, Eichelberger MR, Gotschall CS, Sivit CJ, Randolph JG (1996). Nonoperative management of blunt hepatic and splenic injury in children. Ann Surg.

